# Dairy Consumption and Cardiometabolic Risk Factors in Patients with Type 2 Diabetes and Overweight or Obesity during Intensive Multidisciplinary Weight Management: A Prospective Observational Study

**DOI:** 10.3390/nu12061643

**Published:** 2020-06-02

**Authors:** Shaheen Tomah, Ahmed H. Eldib, Mhd Wael Tasabehji, Joanna Mitri, Veronica Salsberg, Marwa R. Al-Badri, Hannah Gardner, Osama Hamdy

**Affiliations:** 1 Research Division, Joslin Diabetes Center, Boston, MA 02215, USA; ahmed.eldib@joslin.harvard.edu (A.H.E.); mhdwael.tasabehji@joslin.harvard.edu (M.W.T.); joanna.mitri@joslin.harvard.edu (J.M.); vshaw83@gmail.com (V.S.); marwa.albadri@joslin.harvard.edu (M.R.A.-B.); hannah.gardner@joslin.harvard.edu (H.G.); 2 Department of Medicine, Harvard Medical School, Boston, MA 02215, USA

**Keywords:** dairy consumption, type 2 diabetes, obesity, weight management

## Abstract

Dairy products are integral parts of healthy diets; however, their association with cardiometabolic (CM) health among patients with type 2 diabetes (T2D) undergoing weight management is debated. We examined the relationship between dairy consumption and CM biomarkers in 45 subjects with T2D and obesity (mean age 56 ± 9 yrs, 40% female) enrolled in a 12-week intensive multidisciplinary weight management (IMWM) program. After the IMWM program (intervention phase), subjects were followed for 12 weeks (maintenance phase). We stratified subjects based on initial average dairy consumption into infrequent (IFR), less-frequent (LFR), and frequent (FR) consumers. Outcomes were assessed at baseline, 12, and 24 weeks. There were no differences between tertiles at baseline except for higher total energy intake among FR compared with IFR. HbA1c changes showed no association with dairy consumption at 12 or 24 weeks. FR Females achieved greater weight loss at 12 weeks compared with IFR peers (−4.5 kg; 95%CI: −5.5, −3.5). There was a trend towards lower HDL-C with increasing dairy consumption during the intervention phase. In subjects with T2D and overweight or obesity, dairy consumption during weight management is not associated with HbA1c changes but with lower HDL-C and with higher magnitude of weight loss among females.

## 1. Introduction

Diabetes is a major public health problem. In 2019, 463 million people globally had diabetes with type 2 diabetes accounting for ~90% of cases [1]. This number is projected to reach 700 million by 2045, accompanied by an exponential growth in the economic burden of diabetes-related complications and an expected expenditure of 845 billion USD [1]. Lifestyle intervention through nutrition therapy and exercise is the cornerstone of type 2 diabetes management [2,3,4].

In an era of precision medicine and individualized nutrition, it is crucial to investigate the impact of certain types of food and nutrients on disease prevention and management. Dairy products such as milk, cheese, and yogurt have been incorporated into many healthy eating plans, as they are rich in protein, calcium, vitamin D, and potassium [5,6]. However, dairy products are also rich in saturated fatty acids (SFAs), dense in calories, and their effects on cardiometabolic (CM) health remain uncertain or controversial [7,8,9,10]. The major controversy is revolved around their effect on glycemia, body weight, and lipid profile. It is thought that adequate dairy intake could facilitate weight loss in the setting of a hypocaloric diet [11,12,13]. Dairy proteins suppress short-term food intake along with increasing satiety and stimulating regulatory mechanisms, which are known to signal satiation and satiety [14]. Evidence from meta-analyses of randomized controlled trials (RCTs) suggested that dairy products may modestly assist weight loss during short-term or energy-restricted studies [11] and suggested that yogurt consumption is associated with a lower risk of obesity [12]. Another meta-analysis suggested that increased dairy consumption as part of energy-restricted diets resulted in greater reduction in body weight and fat mass without excessive loss of lean body mass in adults [13]. However, other studies showed no effect of high dairy consumption on body composition or lipid profile among adults with obesity [15]. Despite several studies in people without diabetes, there is a paucity of data regarding the relationship between dairy consumption and CM risk factors in patients with type 2 diabetes and overweight or obesity during intensive multidisciplinary weight management (IMWM).

In the current study, we investigated whether higher dairy consumption during a 12-week IMWM program in patients with type 2 diabetes would be associated with improvements in HbA1c, body weight, and other established CM risk factors for up to 24 weeks of follow-up.

## 2. Materials and Methods

Details about our IMWM program were previously published in several papers [16,17,18] Briefly, the Weight Achievement and Intensive Treatment (Why WAIT) is a 12-week IMWM program for weight reduction and intensive diabetes management designed for real-world clinical practice and implemented at a tertiary care center in Boston, MA, USA since 2005. The program comprises intensive medication adjustments, structured dietary intervention, customized individual and group exercise plans, and cognitive behavioral therapy. For more details on the IMWM, please see Supplementary Material.

### 2.1. Study Design and Subjects

This is a prospective cohort observational clinical study of adults with overweight or obesity and type 2 diabetes with HbA1c > 7% on stable doses of antihyperglycemic medications for at least three months preceding the screening. The institutions’ Committee on Human Studies approved the study protocol, and participants signed informed consent forms before enrollment. This study was registered at clinicaltrials.gov (NCT02896140). The study protocol was conducted following the principles described in the Declaration of Helsinki.

Eligible subjects for this study were patients diagnosed with type 2 diabetes for ≥ 3 months, aged 18 to 75 years old, with a body mass index (BMI) ≥ 25 kg/m^2^, and enrolled in the IMWM program. Exclusion criteria included pregnancy, lactose intolerance, cow milk allergy, recent cardiovascular event (e.g., myocardial infarction, stroke) ≤ 6 months before the screening visit, history of congestive heart failure, active malignancy (excluding carcinoma-in-situ of the cervix and the following dermal malignancies: basal cell carcinoma, and squamous cell carcinoma), and chronic gastrointestinal disease (e.g., Crohn’s disease, ulcerative colitis, chronic malabsorption, chronic diarrhea, and gastroparesis).

**Figure 1:**
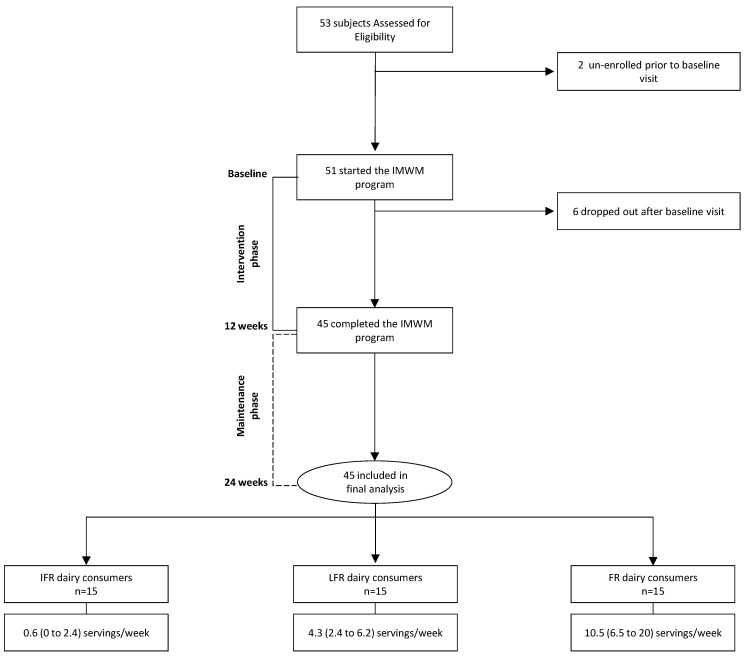
Flow of study subjects stratified by tertiles of weekly average dairy consumption during the IMWM program (IFR, LFR, and FR). Data are mean (range). Abbreviations: IMWM, intensive multidisciplinary weight management; IFR, infrequent; LFR, less-frequent; FR, frequent.

Figure 1 shows the study flow. We enrolled 53 subjects with type 2 diabetes and overweight or obesity that were accepted into the 12-week IMWM program. Two subjects unenrolled after screening and six dropped-out after baseline visit and were excluded from the analysis.

During the 12-week IMWM program (intervention phase), participants received weekly group education. The intervention phase was followed by a 12-week maintenance phase to monitor the progression of the study outcomes.

According to the average dairy consumption during the intervention phase, we stratified the study cohort into tertiles: infrequent (IFR), less-frequent (LFR), and frequent (FR) dairy consumers. Clinical and laboratory data were evaluated at baseline, 12, and 24 weeks.

### 2.2. Assessment of Dietary Intake

At the beginning of the study, participants were instructed to record their daily intake of dairy products in a dedicated dairy logbook. In addition, study participants were instructed to record their total food intake for three days in a designated logbook before each study visit (baseline, 12, and 24 weeks). During visits, the registered dietitian (RD) reviewed the three-day food logbook with each participant to reconcile any missing data. In addition to the three study visits (baseline, 12 and 24 weeks), each participant received two follow-up phone calls from the study RD during the maintenance phase, at weeks 16 and 20, to answer any questions and to improve adherence to dietary logging. Changes in TEI and dietary macronutrients were assessed by analyzing three-day food logs collected at baseline, 12, and 24 weeks using Food Processor Diet & Nutrient Analysis Software (version 11.1.620, 2015, ESHA Research, Salem, OR, USA). Dairy serving size was defined as 8 fluid ounces (–237 mL) of milk, 8 fluid ounces of yogurt, and 1.5 ounces (–42.5 g) of hard cheese (e.g., cheddar, Swiss, etc.) or 2 ounces (–56.7 g) of processed cheese (e.g., American cheese); each count as 1 serving of dairy [19]. Consumption of butter, dairy-based desserts such as ice cream, sour cream, cream cheese, cream, and half-and-half did not count as servings of dairy products.

### 2.3. Assessment of Clinical and Laboratory Parameters

Subjects were instructed to fast overnight prior to the baseline, 12, and 24 weeks study visits. Anthropometric measurements and venous blood samples were collected at each visit following standard protocol. HbA1c, fasting plasma glucose, and CRP were analyzed by immunoturbidimetric assay (instruments C513, C701, and 502, respectively, Roche Diagnostics). Serum insulin was analyzed by electrochemiluminescent immunoassay (ECLIA, instrument E602, Roche Diagnostics). Lipid profile was analyzed by enzymatic colorimetric assay (instrument C702, Roche Diagnostics). Blood pressure was measured in the seated position using CARESCAPE™ V100 monitor (GE medical technologies, Milwaukee, WI, USA). Body weight was measured using a calibrated scale (Tanita BWB–800, Tokyo, Japan). Body composition measurements were performed using a professional version of bioelectrical impedance analyzer (Tanita TBF–215, Tokyo, Japan). Visceral fat was measured using a validated bioelectrical impedance device (Tanita, Viscan AB–140, Tokyo, Japan) and was expressed in arbitrary units ranging from 1 to 59. Height was measured without shoes. Waist circumference was measured just above the hip bone, and hip circumference was measured around the maximum circumference of the buttocks. Insulin sensitivity was calculated using the homeostatic model assessment of insulin resistance (HOMA-IR) equation from fasting plasma glucose and serum insulin at baseline and 12 and 24 weeks [20].

### 2.4. Study Outcomes

We examined the relationship between dairy consumption and glycemic control, body weight, lipid profile, and other CM risk factors among patients with type 2 diabetes and obesity during an IMWM program (intervention phase), and whether dairy consumption frequency is associated with maintenance of weight loss and/or other CM risk factors during the maintenance phase.

The primary outcome of this study is to investigate whether higher dairy consumption for the first 12 weeks is related to improvement in HbA1c and body weight at 12 and 24 weeks. Secondary outcomes included the association between frequency of dairy consumption and changes at 12 and 24 weeks in % total energy intake (TEI) from fat, saturated fat, carbohydrates, and protein; body composition parameters; total cholesterol; LDL-C; HDL-C; VLDL-C; triglycerides; waist and hip circumferences; fasting glucose; insulin sensitivity; CRP; and systolic and diastolic blood pressure (BP).

### 2.5. Statistical Analysis

All study-related quantitative, qualitative, and clinical data were collected and managed using REDCap (Research Electronic Data Capture) [21]. Demographic and baseline characteristics were evaluated using descriptive statistics. Continuous variables are reported as mean ± standard deviation (SD)/ standard error of measurement (SEM) or mean ± 95% confidence intervals (CI) as appropriate for their distribution, as determined by the Shapiro–Wilk test. Categorical variables are presented as percentages. Descriptive characteristics of dairy consumption tertiles were compared using one-way analysis of variance (ANOVA) for continuous variables, and Pearson’s chi-squared test for categorical variables. A nonparametric test for trend across ordered groups, an extension of the Wilcoxon rank-sum test, was used to explore the relationship between tertiles of dairy consumption and change in clinical outcomes followed by step-wise univariate and multivariable linear regression models adjusted for candidate covariates to investigate the association between average dairy consumption and selected outcomes that showed a trend in the initial analysis (changes in HDL-C, and VLDL-C). Subgroup analyses by sex were performed using one-way ANOVA followed by Bonferroni correction for multiple comparisons. A two-sided *p*-value of <0.05 was considered statistically significant. Statistical analysis was conducted using STATA Special Edition 15.0 for Windows (StataCorp, College Station, TX, USA 2017).

## 3. Results

### 3.1. Characteristics of Study Subjects

Table 1 shows the baseline demographic and dietary characteristics of study subjects stratified by tertiles of average dairy consumption. There were no differences between tertiles of dairy consumption at baseline except for higher TEI in FR compared with the IFR dairy consumers (2106 ± 498 kcal/day vs. 1496 ± 540 kcal/day, respectively, *p* = 0.008).

### 3.2. Dairy Consumption and its Fat Content among Study Subjects during the Intervention and Maintenance Phases

**Figure 2:**
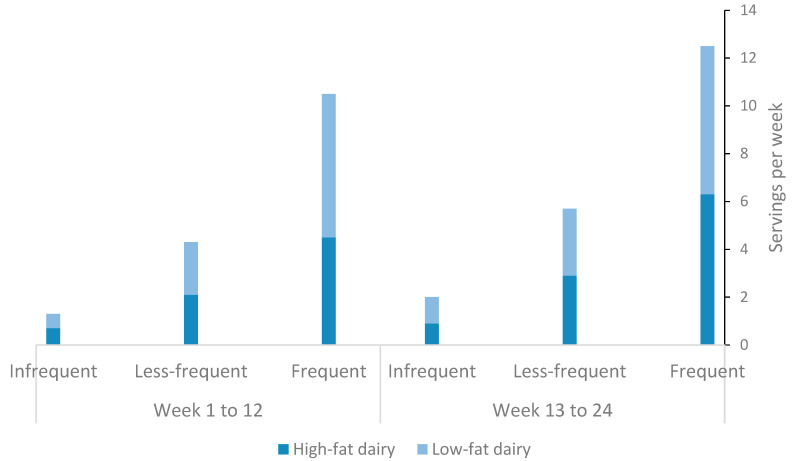
Weekly average dairy consumption and its fat content among study subjects during the intervention phase (week 1 to 12) and the maintenance phase (week 13 to 24).

Average weekly consumption of dairy products was 10.5 (6.5 to 20) servings per week in FR consumers compared with 4.3 (2.4 to 6.2) servings per week and 0.6 (0 to 2.4) servings per week in LFR and IFR consumers, respectively. All three tertiles consumed on average more servings of dairy during the maintenance phase, albeit, the increase in dairy consumption was statistically significant only in LFR dairy consumers (+33%, 4.3 servings/week at 12 weeks vs. 5.6 servings/week at 24 weeks, *p* < 0.05). Consumption of low- and high-fat dairy products was balanced within each tertile (Figure 2).

### 3.3. Changes in Study Outcomes from Baseline to 12 Weeks (Intervention Phase) and 24 Weeks (Maintenance Phase)

#### 3.3.1. Changes in TEI and Macronutrients

Table 2 shows the changes in dietary and clinical parameters during the intervention and the maintenance phases among study subjects. At 12 weeks, TEI in LFR decreased by −312 kcal at 12 weeks (95% CI: −596, −28 kcal, *p* < 0.05) and was maintained at −73 kcal (95% CI: −394, 247 kcal) at 24 weeks. In FR, TEI was decreased by −379 kcal at 12 weeks (95% CI: −760, 2.7 kcal) and −209 kcal (95% CI: −574, 155 kcal) at 24 weeks. While in IFR, TEI was decreased by −123 kcal at 12 weeks (95% CI: −500, 254 kcal) and increased by 30 kcal at 24 weeks (95% CI: −352, 413 kcal). FR dairy consumers increased their %TEI from protein at 12 weeks by 5.8% (95% CI: 1.1, 10.6%, *p* < 0.05) compared with 2.4% (95% CI: −1.4, 6.2%) in LFR consumers and 2.3% (95% CI: −1.9, 6.4%) among IFR consumers (*p* for trend = 0.06).

#### 3.3.2. Changes in HbA1c, Body Weight, and Body Composition Parameters

HbA1c decreased in all three tertiles at 12 and 24 weeks compared to baseline but showed non-significant relation to dairy consumption (Table 2).

**Figure 3:**
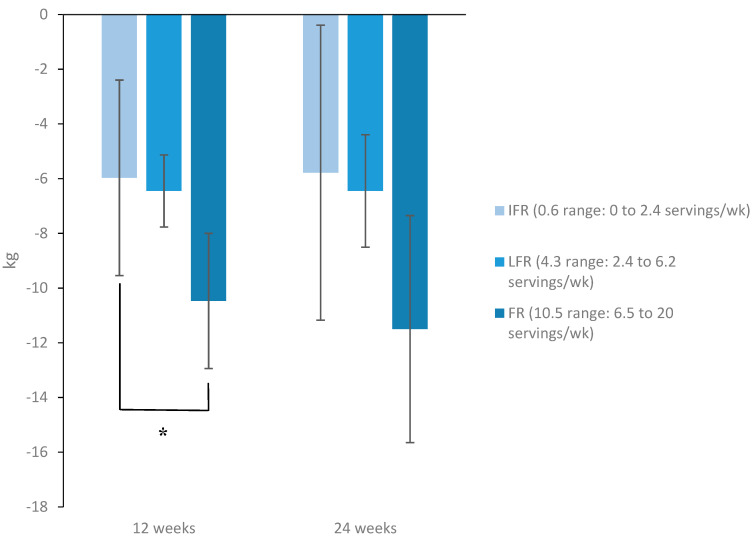
Changes in body weight at the end of the intervention phase (12 weeks) and the end of the maintenance phase (24 weeks) among female subjects stratified by dairy consumption. Female subjects who consumed, on average, 10.5 (6.5 to 20) servings/wk of dairy products (*n* = 7) during the IMWM program achieved a significantly higher amount of weight loss at 12 weeks compared with peers who consumed, on average, 0.6 (0 to 2.4) servings/wk (*n* = 7). *p = 0.027 for FR vs. IFR. Analyses were done using one-way ANOVA followed by Bonferroni correction for multiple comparisons. These differences remained significant after adjusting for baseline body weight, TEI, fat mass, and age. At 24 weeks, there was no significant difference in body weight change between tertiles. Data are mean ± SEM. Abbreviations: FR, frequent dairy consumers; LFR, less-frequent dairy consumers; IFR, infrequent dairy consumers.

Body weight decreased significantly in the total cohort at 12 weeks by −8.6 kg (95% CI: −16.5, −0.7 kg, *p* = 0.03). Body weight maintenance at 24 weeks was −10 kg (95% CI: −24.6, 4.7 kg) in FR compared with −9.5 kg (95% CI: −24.2, 5.3 kg) and −7.6 kg (95% CI: −19.4, 4.2) in LFR and IFR, respectively (*p* for trend = 0.23) (Table 2). Changes in body weight and body composition parameters were not associated with dairy consumption in the total cohort. In subgroup analyses by sex, weight loss at 12 weeks was significantly higher among FR females (*n* = 7) compared with IFR peers (*n* = 7) (−10.5 kg, 95% CI: −12.8, −8.2 vs. −6 kg, 95% CI: −9.3, −2.7; *p* = 0.027). These differences remained significant after adjusting for baseline body weight, TEI, fat mass, and age. However, at 24 weeks, FR females maintained a weight loss of −11.5 kg (95% CI: −15.3, −7.7) compared with −5.8 kg (95% CI: −17.8, 6.3) for peers in IFR dairy consumers vs.; *p* = 0.06) (Figure 3).

#### 3.3.3. Changes in Markers of Inflammation, Glycemia, and BP

Changes in CRP, fasting glucose, HOMA-IR, and systolic or diastolic BP showed no relation to dairy consumption at 12 or 24 weeks (Table 2).

#### 3.3.4. Changes in Lipid Profile

At the end of the intervention phase, HDL-C decreased by 2 mg/dL (95% CI: −10, 6 mg/dL) in FR compared with an increase of 3 mg/dL (95% CI: −8, 14 mg/dL) and 2 mg/dL (95% CI: −8, 12 mg/dL) in LFR and IFR consumers respectively (p for trend = 0.03). Moreover, the association between change in HDL-C and dairy consumption remained significant after adjusting for baseline HDL-C, TEI, fat mass, age, and sex (β = −3.4; 95% CI: −6.2, −0.7; *p* = 0.015) (Table 3).

At the end of the maintenance phase, VLDL-C decreased by 3 mg/dL (95% CI: −11, 5 mg/dL) in FR compared with a decrease of 2 mg/dL (95% CI: −14, 10 mg/dL) and 18 mg/dL (95% CI: −35, −1 mg/dL, *p* < 0.05 compared to baseline) in LFR and IFR dairy consumers respectively (p for trend = 0.04). However, after adjusting for baseline VLDL-C, the relationship between change in VLDL-C and dairy consumption was no longer significant (β = 2.4; 95% CI: −1.8, 6.7; *p* = 0.25) (Table 3).

Changes in total cholesterol LDL-C and Triglycerides showed no association with dairy consumption during the intervention or the maintenance phases.

## 4. Discussion

Our study showed that frequency of dairy consumption during 12 weeks of IMWM program in patients with type 2 diabetes and overweight or obesity is not associated with changes in HbA1c. Several mechanisms may explain the absence of a negative association between frequent dairy consumption and metabolic health. Higher dairy intake was positively associated with systemic and hepatic insulin sensitivity and a more favorable oral glucose tolerance test [22]. Dairy amino acids, such as lysine and branched-chain amino acids (BCAAs)—leucine, isoleucine, and valine—may enhance insulin secretion and potentially decrease postprandial glycemia [23]. Moreover, previous reports suggested that dairy-derived fatty acids may be associated with favorable metabolic outcomes [24,25]. A cohort study found that serum phospholipid trans-palmitoleic acid (trans 16:1n-7), was negatively associated with insulin resistance [25]. In another cohort study, dairy fat consumption was inversely associated with incidence of diabetes [26] as measured by circulating biomarkers of dairy fat, including trans-16:1n-7, pentadecanoic acid (15:0), and heptadecanoic acid (17:0).

In our cohort, frequency of dairy consumption was associated with changes in body weight at 12 weeks only among female subjects. In subgroup analyses by sex, we found that females in the highest tertile of dairy consumption (FR) who consumed 10.5 servings/week (range: 6.5 to 20 servings/week) achieved significantly greater weight loss at 12 weeks compared with peers in the lowest tertile (IFR) who consumed 0.6 servings/week (range: 0 to 2.4 servings/week). However, at 24 weeks, the difference in weight loss between tertiles was not significant. This observation may be explained by the fact that dairy proteins may suppress short-term food intake along with increasing satiety, which may have aided weight loss [14]. However, it is unclear why females may have responded differently to increased dairy intake. These observations are in line with findings from a meta-analysis of RCTs where increased dairy consumption as part of energy-restricted diets resulted in greater reduction in body weight and fat mass, without excessive loss of lean body mass in studies of predominantly female subjects (90%) aged 18–50 years. These effects were absent in the incorporated studies that included resistance training [13] like ours. The assumption is that incorporating resistance training, which minimizes lean mass loss, minimizes the effect of dairy consumption on total body weight. Maintenance of weight loss in our cohort at 24 weeks was −10, −9.5, and −7.6 kg among FR, LFR, and IFR dairy consumers, respectively, with no significant differences between tertiles (Table 2). Our observations are consistent with the findings seen in a meta-analysis of cohort studies where dairy consumption was not associated with changes in body weight [12] and with another meta-analysis of 29 RCTs, where no significant difference in body weight was observed between the dairy intervention group and the control group (−0.14 kg; 95% CI: −0.66, 0.38 kg) [11]. However, in a subgroup analysis of studies that incorporated energy-restriction, there was a significant reduction in body weight in the dairy intervention group compared to the control (−0.79 kg; 95% CI: −1.35, −0.23 kg; I2 = 38.5%). Similarly, dairy consumption significantly reduced body weight in short-term interventions of less than 12 months (−0.47 kg; 95% CI: −0.90, −0.03 kg; I2 = 59.2%), while long-term interventions of more than a year resulted in non-significant changes (0.66 kg; 95% CI: −0.14, 1.46 kg; I2 = 80.7%) [11]. Although our intervention was of short-term and incorporated energy-restriction, the observed difference in body weight outcome with increasing dairy consumption in our cohort was sex-dependent.

During the maintenance phase, subjects consumed on average more servings of dairy per week, albeit, the increase in dairy consumption was statistically significant only in LFR dairy consumers (+33%, Figure 2.). This observation may be explained by relaxed energy restrictions following the commencement of the IMWM program.

In our intervention cohort, there was a trend toward lower HDL-C at the end of the 12-week IMWM program with increased frequency of dairy consumption. Furthermore, this inverse association remained significant after adjusting for baseline HDL-C, TEI, fat mass, age, and sex (see Table 3). Initially, there was a trend toward lower VLDL-C with decreased frequency of dairy consumption during the maintenance phase. However, in our adjusted model, this trend was attenuated after adjusting for baseline VLDL-C levels, which was numerically higher among IFR compared with LFR and FR dairy consumers (see Table 1). Moreover, this trend was further weakened after adjusting for TEI, fat mass, age, and sex (see Table 3).

In this study, we observed no association between frequency of dairy consumption and markers of inflammation, glycemia, BP, total cholesterol, LDL-C, or triglycerides either during the 12-week IMWM program or during the 12-week maintenance phase.

Our study has several limitations, including the small sample size, lack of control group, and being conducted at a single tertiary care facility. Although participants were not directly observed while consuming dairy products, each participant completed a daily logbook to report accurate dairy intake, and follow-up phone calls with a registered dietitian encouraged better adherence. It is important to note that dairy products largely differ by their fat and carbohydrate content. Although consumption of low- and high-fat dairy was balanced within each tertile in our cohort, different dairy products may exert heterogeneous effects on CM health. It is also important to recognize that apart from dairy consumption, modification of dietary habits such as calorie replacements, as part of a dietary intervention, with more carbohydrates or protein might affect blood glucose levels. Lastly, our IMWM program requires considerable time commitment and financial cost—reimbursable by most insurance plans—which may limit the generalizability of our findings.

## 5. Conclusions

In conclusion, this study suggests that, in the context of a 12-week IMWM program, increased frequency of dairy consumption in patients with type 2 diabetes and overweight or obesity is not associated with changes in HbA1c for up to 24 weeks of follow-up, but with a greater magnitude of weight loss at 12 weeks only among female subjects, which warrants further investigation. Frequent dairy consumption was inversely associated with serum HDL-C levels after adjusting for other covariates. Randomized controlled studies with larger sample sizes and for longer duration of intervention are needed to investigate whether addition of dairy products as part of meal plans has any impact on CM health during weight management programs in patients with type 2 diabetes and overweight or obesity.

## Figures and Tables

**Table 1:** Baseline demographic, dietary, and clinical characteristics of study subjects with type 2. diabetes and overweight or obesity stratified by frequency of dairy consumption.

	All Subjects (*N* = 45)	IFR (*n* = 15)	LFR (*n* = 15)	FR (*n* = 15)	*P* Value ^1^
Age (years)	56 (9)	57 (7)	58 (9)	53 (10)	0.3
Female sex (%)	40%	46.7%	26.7%	46.7%	0.5
Race/ethnicity (%)					
Non-Hispanic White	82%	66.7%	92.9%	84.6%	0.2
African American	12.8%	25%	7.1%	7.7%	0.3
Asian	0%	0%	0%	0%	-
Hispanic	2.6%	0%	0%	7.7%	0.3
Other/Not reported	2.6%	8.3%	0%	0%	0.3
Diabetes duration (years)	10.9 (7.1)	11 (6)	12 (7)	9 (8)	0.7
Total energy intake (kcal/d)	1880 (544)	1496 (540)	1962 (448)	2106 (498)	0.008
Energy from total fat (%)	37.5 (5.6)	37.4 (4.4)	36.2 (6.1)	38.8 (5.8)	0.9
Energy from saturated fat (%)	11.8 (3)	11.2 (2.4)	11 (2.9)	13.1 (3.2)	0.1
Energy from carbohydrate (%)	40.3 (6.5)	41.3 (5.8)	40 (5.8)	39.9 (7.7)	0.8
Energy from protein (%)	20.7 (5)	21 (5.5)	20.8 (4.3)	20.2 (5.6)	0.9
Body weight (kg)	106.3 (19.1)	102.3 (15.9)	102.4 (20.6)	114.1 (19.3)	0.15
Body Mass Index (kg/m^2^)	36 (5)	36 (4)	35 (6)	37 (5)	0.5
HbA1c (%)	7.9 (1.3)	8 (1.4)	7.6 (1.2)	8.1 (1.4)	0.6
Total Cholesterol (mg/dL)	162 (35)	165 (33)	156 (31)	167 (41)	0.7
HDL-Cholesterol (mg/dL)	46 (12)	48 (12)	44 (14)	45 (11)	0.7
LDL-Cholesterol (mg/dL)	85 (30)	83 (22)	80 (28)	91 (38)	0.6
VLDL-Cholesterol (mg/dL)	32 (21)	40 (30)	28 (15)	27 (9)	0.2
Triglycerides (mg/dL)	173 (145)	165 (88)	167 (105)	187 (217)	0.9
Waist circumference (cm)	120 (13)	119 (8.3)	117 (13)	124 (16)	0.3
Hip circumference (cm)	120.7 (14)	120 (7.6)	116 (16)	126 (15.7)	0.2
Waist/hip ratio	1 (0.07)	1 (0.07)	1 (0.07)	1 (0.07)	0.6
Body fat (%)	40.1 (8)	41.8 (7.6)	37.7 (7.4)	40.9 (9)	0.4
Fat mass (kg)	38 (15)	38.8 (11.7)	34 (13)	41 (19)	0.4
Fat free mass (kg)	57.2 (20.4)	55.3 (18)	57 (19.3)	59 (24.4)	0.9
Total body water (kg)	42 (15)	40.5 (13.2)	42 (14.2)	43.3 (18)	0.9
Trunk fat (%)	46.2 (8.4)	45 (6.5)	44 (9.9)	50 (8)	0.2
Visceral fat (arbitrary units)	20.2 (8.6)	18.9 (7.1)	20.8 (9)	21 (10.4)	0.8
CRP (mg/L)	4.3 (6.7)	2.6 (2.2)	3.5 (3.6)	6.6 (10.7)	0.2
Fasting glucose (mg/dL)	152 (51)	147 (54)	143 (42)	166 (56)	0.4
HOMA-IR	6.9 (5.6)	7.7 (6.6)	6.1 (5.4)	7.1 (4.8)	0.7
Systolic blood pressure (mmHg)	128 (16)	124 (12)	130 (16)	131 (21)	0.45
Diastolic blood pressure (mmHg)	72 (10)	70 (9)	74 (12)	72 (9)	0.56
Diabetes medications (%)					
Metformin	84.4%	86.7%	73.3%	93.3%	0.3
SFUs	26.6%	20%	33.3%	26.7%	0.7
DPP-4 inhibitors	4.4%	0%	6.7%	6.7%	0.6
SGLT-2 inhibitors	26.6%	26.7%	26.7%	26.7%	1
GLP-1 analogues	37.7%	33.3%	53.3%	26.7%	0.29
TZDs	2.2%	0%	0%	6.7%	0.36
Other	44.4%	26.7%	53.3%	53.3%	0.23

Data are mean (SD) or %. ^1^ One-way ANOVA or Pearson’s Chi-squared test. Abbreviations: FR, frequent dairy consumers; LFR, less-frequent dairy consumers; IFR, infrequent dairy consumers; CRP, c-reactive protein; HOMA-IR, homeostatic model of insulin resistance; SFUs, sulfonylureas; DPP, dipeptidyl peptidase; SGLT, sodium-glucose transport proteins; GLP, glucagon-like-peptide; TZDs, Thiazolidinedione.

**Table 2:** Changes from baseline in dietary intake and cardiometabolic parameters at the end of intervention phase (12 weeks) of intensive medical weight management and at the end of maintenance phase (24 weeks) among patients with type 2 diabetes and overweight or obesity stratified by frequency of dairy consumption.

	IFR (*n* = 15)		LFR (*n* = 15)		FR (*n* = 15)		*P* ^3^	*P* ^4^
	12 Weeks	24 Weeks	12 Weeks	24 Weeks	12 Weeks	24 Weeks		
Total energy intake (kcal/d)	−123 (−500, 254)	30 (−352, 413)	−312 (−596, −28) ^1^	−73 (−394, 247)	−379 (−760, 2.7)	−209 (−574, 155)	0.4	0.6
Energy from total fat (%)	−10 (−29, 9)	−2 (−21, 18)	−11 (−30, 7)	−6 (−22, 10)	−19 (−42, 4)	−9 (−31, 14)	0.4	0.6
Energy from saturated fat (%)	−1.8 (−3.8, 0.3)	−0.3 (−2.2, 1.5)	−0.8 (−2.7, 1)	−0.6 (−2.6, 1.4)	−1 (−3.5, 1.5)	−0.4 (−3.1, 2.4)	0.3	0.9
Energy from carbohydrate (%)	0.9 (−4, 5.9)	1.4 (−3.2, 6)	−1 (−5.6, 3.6)	−1.1 (−5.8, 3.5)	−2.7 (−8.4, 3)	−2.5 (−7.7, 2.7)	0.4	0.5
Energy from protein (%)	2.3 (−1.9, 6.4)	0.2 (−3.8, 4.1)	2.4 (−1.4, 6.2)	1.2 (−2.1, 4.6)	5.8 (1.1, 10.6)^1^	3.6 (−0.6, 7.8)	0.1	0.06
Body weight (kg)	−8 (−19.4, 3.6)	−7.6 (−19.4, 4.2)	−8.3 (−23.6, 7)	−9.5(−24.2, 5.3)	−9.5 (−24, 5)	−10 (−24.6, 4.7)	0.3	0.23
Body Mass Index (kg/m^2^)	−2.8 (−5.8, 0.3)	−2.6 (−5.8, 0.6)	−3 (−8, 2.1)	−3.3 (−8.3, 1.6)	−3.1 (−6.7, 0.5)	−3.3 (−7, 0.5)	0.4	0.32
HbA1c (%)	−1.32 (−2.2, −0.5) ^2^	−1.13 (−2, −0.3) ^1^	−1.12 (−1.84, −0.4) ^2^	−0.64 (−1.65, 0.37)	−1.48 (−2.3, −0.6) ^2^	−1.18 (−2, −0.34) ^2^	0.8	0.6
Lipid profile								
Total Cholesterol (mg/dL)	−13 (−38, 12)	−10 (−31, 12)	−16 (−39, 6)	−5 (−30, 19)	−20 (−50, 9)	−11 (−40, 18)	0.5	0.6
HDL−C (mg/dL)	2 (−8, 12)	6 (−4, 15)	3 (−8, 14)	6 (−5, 17)	−2 (−10, 6)	2 (−7, 11)	0.03	0.09
LDL−C (mg/dL)	−6 (−24, 11)	−4 (−19, 10)	−11 (−30, 8)	−6 (−26, 14)	−15 (−41, 12)	−7 (−31, 18)	0.6	0.9
VLDL−C (mg/dL)	−16 (−34, 1)	−18 (−35, −1) ^1^	−5 (−15, 5)	−2 (−14, 10)	−1 (−10, 8)	−3 (−11, 5)	0.06	0.04
Triglycerides (mg/dL)	−48 (−106, 11)	−58 (−111, −4) ^1^	−52 (−115, 10)	−37 (−108, 33)	−58 (−182, 66)	−69 (−192, 54)	0.2	0.15
Waist circumference (cm)	−7.8 (−15, −0.4) ^1^	−7.5 (−15, −0.3) ^1^	−9 (−19, 1)	−9.6 (−20, 0.5)	−6.5 (−18, 5)	−8 (−21, 5)	0.5	0.9
Hip circumference (cm)	−7 (−13, −0.6) ^1^	−5 (−11, 2)	−7 (−19, 5)	−8 (−20, 3.6)	−9 (−20, 1,7)	−6.5 (−19, 6)	0.7	0.7
Waist/hip ratio	−0.01 (−0.07, 0.05)	−0.02 (−0.09, 0.04)	−0.02 (−0.07, 0.03)	−0.01 (−0.07, 0.04)	0.02 (−0.03, 0.07)	−0.02 (−0.08, 0.04)	0.2	0.9
Body fat (%)	−4.3 (−11, 2.5)	−4.5 (−11, 1.8)	−4.1 (−10.4, 2.1)	−4.2 (−10.6, 2.1)	−2.6 (−8.7, 3.6)	−2.2 (−8.4, 3.4)	0.4	0.2
Fat mass (kg)	−5 (−14.6, 4.6)	−3.7 (−12.5, 5.1)	−1.5 (−11.8, 8.7)	−1.9 (−12.2, 8.3)	−0.73 (−12.7, 11.2)	−0.4 (−12.5, 11.8)	0.3	0.3
Fat free mass (kg)	−2 (−15, 10.8)	3.2 (−10.5, 17)	4.8 (−7.5, 17)	4.1 (−8, 16.2)	5.5 (−9.5, 20.5)	4.8 (−10.2, 19.8)	0.5	0.4
Total body water (kg)	−1.5 (−10.9, 8)	2.4 (−7.7, 12.4)	3.5 (−5.5, 12.5)	3 (−5.9, 12)	−4 (−7, 15)	3.5 (−7.5, 14.5)	0.5	0.4
Trunk fat (%)	−3 (−8.4, 2.3)	−3.8 (−9.2, 1.5)	−8.3 (−17, 0.3)	−7.4 (−16.5, 1.6)	−5.4 (−12.2, 1.4)	−7.1 (−14.3, 0.2)	0.4	0.6
Visceral fat (arbitrary units)	−2.4 (−7.7, 2.9)	−2.1 (−7.7, 3.4)	−4.4 (−11, 2.1)	−3.6 (−10.7, 3.4)	−1.8 (−9.2, 5.6)	−2 (−9.5, 5.6)	0.4	0.5
CRP (mg/L)	−0.9 (−2.3, 0.6)	−0.2 (−1.9, 1.4)	−0.7 (−3.4, 2)	0.1 (−3.1, 3.2)	0.3 (−8.1, 8.7)	−0.3 (−10, 9.3)	0.2	0.6
Fasting glucose (mg/dL)	−24 (−60, 12)	−17 (−53, 17)	−12 (−38, 14)	3 (−35, 41)	−36 (−72, −1) ^1^	−38 (−72, −4) ^1^	0.2	0.4
HOMA−IR	−3.6 (−7.6, 0.4)	−3.2 (−7.2, 0.9)	−1 (−5.3, 3.4)	−0.6 (−5.2, 3.9)	−1.4 (−4.5, 1.6)	−1.8 (−5.4, 1.7)	0.3	0.6
Systolic blood pressure (mmHg)	−3 (−14, 7)	1 (−7, 10)	−7 (−18, 4)	−8 (−21, 5)	−5 (−20, 10)	−6 (−22, 10)	0.9	0.4
Diastolic blood pressure (mmHg)	−1 (−6, 6)	2 (−4, 9)	−6 (−13, 3)	−7 (−15, 1)	−1 (−8, 5)	−1 (−8, 6)	0.6	0.3

Data are mean (95% CI). ^1^ *P*<0.05 for student’s *t*-test compared to baseline; ^2^ *P* < 0.01 for student’s *t*-test compared to baseline; ^3^ *P* for trend at the end of the intervention phase using a nonparametric test for trend (an extension of the Wilcoxon rank-sum test) among ordered groups of dairy consumption; ^4^ *P* for trend at the end of the maintenance phase using a nonparametric test for trend (an extension of the Wilcoxon rank-sum test) among ordered groups of dairy consumption; Abbreviations: FR, frequent dairy consumers; LFR, less-frequent dairy consumers; IFR, infrequent dairy consumers; CRP, c-reactive protein; HOMA-IR, homeostatic model of insulin resistance.

**Table 3:** Association between serum HDL-C and VLDL-C, and dairy consumption.

HDL-C ^1^	Crude		Model 1		Model 2		Model 3	
	β (95%CI)	*p*	β (95%CI)	*p*	β (95%CI)	*p*	β (95%CI)	*p*
Dairy (servings per week)	−1.93 (−4, 0.2)	0.07	−3 (−5.5, −0.5)	0.022	−3.4 (−6, −0.7)	0.013	−3.4 (−6.2, −0.7)	0.015
**VLDL-C ^2^**	**Crude**		**Model 1**		**Model 2**		**Model 3**	
	**β (95%CI)**	***p***	**β (95%CI)**	***p***	**β (95%CI)**	***p***	**β (95%CI)**	***p***
Dairy (servings per week)	6.1 (−0.2, 12.5)	0.058	2.4 (−1.8, 6.7)	0.25	2.8 (−1.7, 7.3)	0.21	2.7 (−1.8, 7.3)	0.22

**^1^** Serum HDL-C levels were inversely associated with frequency of dairy consumption at 12 weeks; Model 1 is adjusted for baseline HDL-C and total energy intake; Model 2 is adjusted for baseline HDL-C, total energy intake, and fat mass; Model 3 is adjusted for baseline HDL-C, total energy intake, fat mass, age, and sex. **^2^** Serum VLDL-C levels were not associated with frequency of dairy consumption at 24 weeks. Model 1 is adjusted for baseline VLDL-C and total energy intake. Model 2 is adjusted for baseline VLDL-C, total energy intake, and fat mass. Model 3 is adjusted for baseline VLDL-C, total energy intake, fat mass, age, and sex. Analyses were done using univariate and multivariable linear regression models adjusted for candidate covariates.

## Supplementary Materials

The following are available online at https://www.mdpi.com/2072-6643/12/6/1643/s1.
