# Supplementary material for: Dairy Consumption and Cardiometabolic Risk Factors in Patients with Type 2 Diabetes and Overweight or Obesity during Intensive Multidisciplinary Weight Management: A Prospective Observational Study
Source: Nutrients. 2020 Jun 2;12(6):1643. doi: 10.3390/nu12061643 (PMC7352424; doi:10.3390/nu12061643)
Supplement: Supplementary file 1 [file nutrients-12-01643-s001.pdf]

The Intensive Multidisciplinary Weight Management (IMWM) program

The Weight Achievement and Intensive Treatment (Why WAIT) is a comprehensive program aimed to improve the well-being of patients with type 2 diabetes and obesity. The program follows a 12-week course of Intensive Multidisciplinary Weight Management (IMWM) that was developed at Joslin Diabetes Center, a tertiary medical center, in 2005. Since then, the Why WAIT program has been conducted by a multidisciplinary team of medical professionals, including a nurse practitioner, a diabetologist, a registered dietitian, an exercise physiologist, clinical psychologists, and behavioral therapists. The program is offered to 12–15 participants per session. The interventions occur weekly in two-hour group sessions. Additionally, participation in the program is covered by most insurance plans. Further context of the four essential components of the program are as follows:

I. **Medication adjustments:** At the beginning of the program, patient's weight gain-promoting medications were substituted when appropriate, with alternative weight neutral or weight loss-promoting medications. Additionally, for patients with an A1C< 7.5%, insulin doses were reduced by 20–30%. However, in patients with an A1C ≥ 7.5%, insulin doses were not altered initially but adjusted during the program when appropriate. Medical evaluations occurred for 30 minutes at weeks 4 and 8 by a nurse practitioner and at week 12 by a diabetologist. Patients

were excluded from the program if they were unable to follow the medication adjustment plans, as to attenuate the risk of severe hypoglycemia during weight loss.

II. **Structured medical nutrition therapy:** A registered dietitian customized hypocaloric meal plans for participants in accordance their sex, age, typical caloric intake, and physical activity. For all meal plans, carbohydrates made up ~40–45% of daily calories, fat <35% with saturated fat < 10%, 1–1.5 g/kg of adjusted body weight from protein, and 14 g of fiber per 1000 calories.

III. **Exercise intervention:** An exercise physiologist (EP) evaluated participants' health status and exercise capacity and used this information to develop personalized exercise plans. Additionally, sixty-minute group exercise sessions were conducted by the EP weekly. Throughout the program, participants were directed to increase the difficulty of their exercise plans. For example, exercise plans that instructed exercise for 20 minutes 4 days/week could be increased to 60 minutes 5–6 days/week.

IV. **Behavioral intervention:** Clinical psychologists and behavioral therapists mainly focused on providing participants with therapeutic methods for weight management during weekly group behavioral support sessions. Their methods applied validated cognitive-behavioral therapeutic approaches for weight loss. (1, 2) Participants were taught behavioral goal-setting, self-monitoring of food intake

and exercise, stimulus control techniques, and relapse prevention. [1,2]

Lastly, the Why WAIT program held weekly education sessions for participants from a medical professional in the program. Each session discussed a relevant topic to the program and offered further expertise on their weight and diabetes managemen

References

[1]Knowler WC, Barrett-Connor E, Fowler SE, Hamman RF, Lachin JM, Walker EA, Nathan DM. Reduction in the incidence of type 2 diabetes with lifestyle intervention or metformin. N Engl J Med. 2002;346(6):393-403.

[2]Wadden TA, Stunkard AJ. Handbook of obesity treatment: Guilford Press; 2002.
